# Effects of environmental condition, size, coat type, and body condition score on rectal temperature prediction in dogs using infrared auricular and surface temperature

**DOI:** 10.14202/vetworld.2022.1314-1322

**Published:** 2022-05-25

**Authors:** Yanisa Lukkanawaraporn, Nutnicha Tiangtas, Vorapan Chaikornkij, Patthamon Nawapakpilai, Sathita Areerat, Pipatpong Chundang, Chalermpol Lekcharoensuk, Attawit Kovitvadhi

**Affiliations:** 1 Faculty of Veterinary Medicine, Kasetsart University, Bangkok 10900, Thailand; 2 Graduate School in Animal Health and Biomedical Science Program, Faculty of Veterinary Medicine, Kasetsart University, Bangkok 10900, Thailand; 3 Department of Physiology, Faculty of Veterinary Medicine, Kasetsart University, Bangkok 10900, Thailand; 4 Department of Companion Animals Clinical Sciences, Faculty of Veterinary Medicine, Kasetsart University, Bangkok 10900, Thailand

**Keywords:** auricular temperature, body surface temperature, dog, infrared surface temperature, rectal surface temperature

## Abstract

**Background and Aim:**

Generally, rectal body temperature (BTrectum) is used to prefer as core body temperature in dogs. However, this procedure is time- and labor-consuming with stress induction. Therefore, infrared auricular temperature (BTear) and surface temperature (ST) could be applied to estimate BTrectum. This study aimed to estimate BTrectum from BTear or ST in various areas and determined the factors that influenced the accuracy of prediction equations.

**Materials and Methods:**

Under controlled temperature (n=197) and ambient temperature (n=183), the parameters BTrectum, BTear, and ST at internal pinna, auricular canal, lateral aspect of shoulder, hip, axillary area, inguinal area, footpad, and anal area (STrectum) were measured. In addition, temperature and humidity levels of the surrounding environment were recorded. The correlation between each measurement technique was calculated. The BTrectum prediction equation was created using all measured data and several influencing factors (environmental condition, size, coat type, and body condition score [BCS]).

**Results:**

The highest correlation with BTrectum was observed for BTear (r=0.61, p<0.01), which was similar to STrectum (r=0.61, p<0.01). Based on multiple linear regression model results using BTrectum as the dependent variable, BTear or STrectum were first selected as independent variables in all estimation equations. Ambient temperatures (R^2^=0.397), small breed (R^2^=0.582), long hair (R^2^=0.418), and/or a BCS of 2 (R^2^=0.557) provided the highest coefficients of determination of the prediction equation.

**Conclusion:**

The most appropriate predictors for estimating BTrectum were STrectum and BTear, which were impacted by the dog’s signalments and the environment. To obtain satisfactory outcomes, the equation must be selected depending on the dog’s signalments and the environmental conditions. However, based on the findings of this investigation, the accuracy remains low in several equations, and further studies are needed to improve the accuracy of the equation, mainly by increasing the sample size and developing a specific equation for each dog’s signaling and environmental condition.

## Introduction

Warm-blooded animals (homeotherms), mammals, and avians need to maintain a constant temperature to ensure normal biochemical and physiological functions [1]. Body temperature (BT) is considered as a common indicator of the physiological and health status in animals, with applications in veterinary medicine, farm management, health monitoring, and animal welfare [2]. Rectal BT is generally used as an indicator of core BT because of the strong correlation between these parameters [3]. However, although this method is commonly performed in real practice, it is time- and labor-consuming [3-5]. To measure rectal temperature, the animal needs to be restrained, leading to stress and, consequently, a higher BT [4,5]. Furthermore, injuries to animals and/or workers can occur [3,4]. In addition, animals suffering from inflammation of the rectum and/or perianal deliver false measurements [3]. Therefore, a mildly invasive and less time-consuming temperature measurement technique is necessary.

Infrared auricular thermometers have been developed to solve problems regarding BT measurements in humans and dogs [2-4,6-8]. Although some studies have reported a strong correlation between rectal and auricular BT in dogs [2,3,6,7], others have found only a slight correlation [4]. Gum temperature can be a good predictor of rectum temperature [8], but this technique, despite its several advantages, still requires close contact to the animals, with the risk of disturbance and disease transmission [5]. Infrared surface temperature (ST), as a non-contact technique with real-time results and minimal time and labor consumption, is measured based on the radiation heat loss in the infrared range [9,10]. Because of its significant benefits, it is widely used in livestock [5,11,12] and companion animals [3,13-15]. Measurement accuracy is greatly influenced by several factors, such as environmental conditions (temperature and humidity) and the location of measurements [3,14]. Moreover, the significant variation in signalments (body condition score [BCS], coat type, breed, and body size) in dogs compared to livestock can lead to inaccurate measurements [3,14,16]. A significant correlation between rectal body and infrared ST in dogs has been reported in several studies, although with considerable variations [3,14]. As the infrared ST generally differs from the rectal BT, a prediction equation is necessary; this equation should be investigated based on signalments in dogs to reduce variation and improve prediction accuracy.

This study aimed to predict rectal temperature in dogs by alternative temperature measurement techniques (auricular body and infrared ST), and accuracy factors were determined.

## Materials and Methods

### Ethical approval

This study was conducted following standard guidelines and was approved by the Institutional Animal Care and Use Committee of Kasetsart University, Bangkok, Thailand (ACKU63-VET-015), with agreement from the dogs’ owners.

### Study period and location

The study was conducted from June to August 2020, which was the summer season, without rain during the entire collection period. The study was conducted at Kasetsart University Veterinary Teaching Hospital, Bangkaen (Faculty of Veterinary Medicine, Kasetsart University, Bangkok, Thailand) and Kasetsart University Veterinary Teaching Hospital, Kamphaengsaen campus (Faculty of Veterinary Medicine, Kasetsart University, Nakhon Pathom, Thailand).

### Animals

There were two experimental groups based on environmental conditions. The controlled temperature environmental group (CT), in an air-conditioned hospital (n=197), was obtained by the systematic random sampling of dogs presented to the Kasetsart University Veterinary Teaching Hospital, Bangkaen. (Faculty of Veterinary Medicine, Kasetsart University, Bangkok, Thailand). The ambient group without air conditioning (AT; n=183) was obtained by systematic random sampling at the Kasetsart University Veterinary Teaching Hospital, Kamphaengsaen campus (Faculty of Veterinary Medicine, Kasetsart University, Nakhon Pathom, Thailand). All dogs in this study were brought for several purposes; dogs with otitis were excluded from the study. The signalments of dogs (BCS, coat types, breed, and breed by size) are represented in Table-1 and were collected by the same observer; BCS and breed by size were evaluated following the recommendations of the American Animal Hospital Association [17] and the American Kennel Club [18], respectively.

**Table 1:** Signalments of dogs (n=380) divided by body condition score, coat types, breed, and breed by size in differences of environmental conditions between control (n=197) and ambient temperature (n=183).

Signalments	Environmental condition		

	Control temperature	Ambient temperature	Total
Body condition score			
1	2	10	12
1.5	0	2	2
2	10	11	21
2.5	26	23	49
3	93	82	175
3.5	28	20	48
4	16	25	41
4.5	2	2	4
5	20	8	28
Coat type			
Short	108	96	204
Long	89	87	176
Breed (Breed by size, AKC, 2020)			
Alaskan Malamute (L)	0	1	1
American Pit Bull Terrier (M)	2	3	5
Beagle (S)	7	6	13
Border collie (M)	1	0	1
Bulldog (M)	5	0	5
Cairn Terrier (S)	0	1	1
Chihuahua (XS)	14	14	28
Chow Chow (M)	0	2	2
Corgi (S)	0	2	2
Crossbreed (Not defined)	57	91	148
Dachshund (S)	1	0	1
French Bulldog (S)	18	0	18
German Shepherd(L)	0	2	2
Golden Retriever (L)	7	9	16
Great Dane (XL)	3	0	3
Labrador Retriever (L)	7	13	20
Maltese (XS)	2	1	3
Miniature Schnauzers (S)	0	1	1
Pomeranian (XS)	24	9	33
Poodle (S)	11	2	13
Pug (S)	3	0	3
Russell Terrier (S)	4	0	4
Shetland Sheepdog (S)	1	0	1
Shih Tzu (S)	13	11	24
Siberian Husky (M)	6	7	13
Thai Bangkaew (M)	7	8	15
West Highland White Terrier (S)	2	0	2
Yorkshire Terrier (XS)	2	0	2
Breed by size (AKC, 2020)			
XS (Toy Breeds)	42	24	66
S (Small breeds)	60	23	83
M (Medium breeds)	21	20	41
L (Large breeds)	14	25	39
XL (Giant breeds)	3	0	3
Crossbreed (Not defined)	57	91	148

### Temperature measurements

BT in two different locations and infrared ST in eight locations was taken after recording the signalments. All temperature measurements were taken on the left side of the dogs. Before the measurements, all dogs were brought indoors, away from sunlight. Rectal BT (BTrectum) was measured by insertion of a digital thermometer (JTMD-201M, Jitron^®^, Jintron Pte Ltd., Zervex, Singapore) about 3 cm into the rectum, attached to the rectal wall. Ear BT (BTear) was measured by an infrared ear thermometer (ThermoScan^®^, Braun GMBH, Kronberg, Germany), which was inserted into the ear canal. ST was measured by an infrared thermal imaging camera which had been adjusted to measure the ST of animals (IR resolution 4800 pixels; UTi85H+, Dongguan Xintai Instrument Co., Ltd., Guangdong, China) at the internal pinna (STpinna), the auricular canal (STear), the lateral aspect of the shoulder (STshoulder), the hip (SThip), the axillary area (STaxillary), the inguinal area (STinguinal), the foot pad (STfootpad), and the anal area (STrectum). The distance between the infrared thermal imaging camera and the measured surface was 30 cm. Environmental temperature (EnvironTemp) and humidity were measured by temperature and humidity data loggers (2C\TEMP-RH, Marathon Products Inc., CA, USA). All measurements were performed by the same researcher and in the same order: BTear, STpinna, STear, STshoulder, SThip, STaxillary, STinguinal, STfootpad, STrectum, and BTrectum. Before measurements were obtained, each animal was given 30 min to acclimate to the room temperature (25±5°C).

### Statistical analysis

All statistical analyses were performed with the R-statistic software in R studio 4.0.2. Differences were considered statistically significant at p<0.05. Descriptive statistics were used to represent all measurement data using the Rcmdr package. The mean values of ambient and control environmental conditions were compared by Student’s t-test in the Rcmdr package as the data showed homogeneity of variance. Pearson’s correlation coefficients were calculated among the measurement techniques by the corrplot package. The stepwise multiple linear regression equation was formulated using the Rcmdr package. The BTrectum served as the dependent variable and the other measurements as independent variables. Scatterplots and Bland-Altman plots of STrectum, BTear, and STrectum versus BTrectum of all measurement data, in the control environmental temperature group and the ambient environmental temperature group were illustrated by the ggplot2 package.

## Results

### Comparison of BT and infrared ST measurements at different environmental conditions

The BTrectum, BTear, and infrared ST values at different environmental conditions and for both experimental groups are shown in Table-2. Most ST values were lower than the BTretum value, except STear, STinguinal, and STrectum at CT. All temperature measurements differed significantly between the groups (p<0.001). The average of all measurement parameters from group CT was lower than that of group AT. The standard deviation of EnvironTemp and humidity in AT was higher than that in CT.

**Table 2:** Rectal, auricular body, and infrared surface temperature in dogs (n=380) in differences environmental condition between control (n=197) and ambient temperature (n=183).

Environmental condition	Parameters		Minimum	Maximum	p-value

	Mean±standard deviation	95% Confidence interval			
Control temperature condition					
BTrectum (°F)	100.2±1.31	100.0-100.4	97.1	102.9	<0.001
BTear (°F)	99.0±1.50	98.7-99.2	93.8	101.8	<0.001
Infrared surface temperature (°F)					
STpinna	94.2±5.87	93.3-95.0	79.5	108.0	<0.001
STear	100.3±3.32	99.8-100.8	86.9	107.0	<0.001
STshoulder	92.1±3.94	91.5-92.6	81.3	102.7	<0.001
SThip	92.5±4.38	91.9-93.2	80.0	110.6	<0.001
STaxillary	99.0±3.86	98.4-99.5	87.0	107.2	<0.001
STinguinal	100.9±3.19	100.5-101.4	83.8	107.7	<0.001
STfootpad	89.9±5.75	89.1-90.7	76.2	102.7	<0.001
STrectum	101.4±2.62	101.0-101.8	93.5	107.0	<0.001
Environment					
EnvironTemp (°F)	83.3±2.43	83.0-83.6	75.9	89.4	<0.001
Humidity (%)	57.0±6.52	56.1-57.9	41.1	74.0	<0.001
Ambient temperature condition					
BTrectum (°F)	100.8±1.50	100.6-101.0	96.3	104.0	-
BTear (°F)	100.2±1.72	99.9-100.4	94.6	104.1	-
Infrared surface temperature (°F)					
STpinna	97.5±5.97	96.6-98.4	81.3	107.2	-
STear	103.4±3.05	103.0-103.9	86.1	108.6	-
STshoulder	93.6±6.22	92.7-94.5	69.7	110.8	-
SThip	94.4±6.14	93.5-95.3	69.2	108.8	-
STaxillary	101.9±4.08	101.3-102.5	84.2	109.5	-
STinguinal	103.9±3.11	103.4-104.3	83.4	109.0	-
STfootpad	93.7±5.86	92.8-94.5	77.0	107.2	-
STrectum	103.5±2.72	103.1-103.9	89.4	109.7	-
Environment					
EnvironTemp (°F)	87.7±3.67	87.1-88.2	79.5	99.7	-
Humidity (%)	62.1±10.5	60.6-63.6	38.2	85.0	-

BT=Body temperature, ST=Surface temperature, EnvironTemp=Environmental temperature

### Correlation among different temperature measurements and environmental conditions

Pearson’s correlation coefficients of the correlation between BTrectum and different measurement techniques in different environmental conditions are shown in Table-3. We observed a significant correlation (p<0.01) between BTrectum and all measured parameters except SThip. Based on all measurement data, significant positive correlations were obtained for BTear (r=0.61, p<0.01) and STrectum (r=0.61, p<0.01). On the one hand, BTear showed the highest correlation with BTrectum at ambient environmental conditions (r=0.63, p<0.01), followed by STrectum (r=0.54, p<0.01). In contrast, STrectum showed the highest correlation with BTrectum at CT (r=0.62, p<0.01), followed by BTear (r=0.51, p<0.01).

**Table 3:** Pearson’s correlation coefficients between rectal body temperature and different temperature measurements in dogs. The information was represented only when a statistically significant difference was established (p<0.01).

Parameters	BTrectum		

	All measurement (n=380)	Environmental condition	

		Control (n=197)	Ambient (n=183)
BTear	0.61	0.51	0.63
STpinna	0.37	0.21	0.44
STear	0.46	0.34	0.51
STshoulder	0.14	-	-
SThip	-	-	-
STaxillary	0.28	0.18	0.25
STinguinal	0.33	0.20	0.33
STfootpad	0.19	-	0.31
STrectum	0.61	0.62	0.54
EnvironTemp	0.31	-	0.27
Humidity (%)	0.23	0.21	-

BT=Body temperature, ST=Surface temperature and EnvironTemp=Environmental temperature

### Prediction equations at different signalments of dogs

The prediction equations with different factors are presented in Table-4. The equations from simple and multiple linear regressions were formulated using BTrectum and other temperature measurements as dependent and independent variables, respectively. The coefficient of determination (R^2^) from all measurements was 0.484; BTear was considered the first predictor, followed by STrectum and STfootpad. The R^2^ from the prediction equation from dogs at AT (R^2^=0.481) was similar to that obtained at CT (R^2^=0.482). The STrectum was the first predictor which was inserted in the equation for CT, whereas BTear was the first predictor for AT.

**Table 4:** The prediction equations on rectal temperature of dogs from different predictors in different environmental conditions, breeds by size, coat types, and body condition scores.

Criteria	No.	Equations	SEE	R^2^
All measurements (n=380)	1	BTrectum=0.504(BTear)+50.29	1.146	0.364
	2	BTrectum=0.334(BTear)+0.197 (STrectum)+47.05	1.040	0.478
	3	BTrectum=0.348(BTear)+0.207 (STrectum)−0.02(STfootpad)+46.47	1.035	0.484
Environmental condition	4	BTrectum=0.310(STrectum)+68.75	1.028	0.386
Control environmental condition (n=197)	5	BTrectum=0.247(STrectum)+0.273(BTear)+48.17	0.960	0.468
	6	BTrectum=0.245(STrectum)+0.289(BTear)−0.028 (STfootpad)+49.21	0.949	0.482
Ambient environmental condition (n=183)	7	BTrectum=0.550(BTear)+45.70	1.169	0.397
	8	BTrectum=0.425(BTear)+0.167(STrectum)+40.99	1.101	0.468
	9	BTrectum=0.390(BTear)+0.128(STrectum)+ 0.073(STear)+41.00	1.090	0.481
Breed by size (n=232)				
Toy Breeds (XS; n=66)	10	BTrectum=0.471(BTear)+53.37	1.121	0.391
Small Breeds (S; n=83)	11	BTrectum=0.334(STrectum)+66.15	0.810	0.582
	12	BTrectum=0.280(STrectum)+0.212(BTear)+50.58	0.769	0.628
Medium Breeds (M; n=41)	13	BTrectum=0.137(STInguinal)+87.00	0.990	0.197
	14	BTrectum=0.111(STInguinal)+0.341(BTear)+55.52	0.936	0.300
	15	BTrectum=0.139(STInguinal)+0.488(BTear)−0.06(STpinna)+43.65	0.894	0.379
Large Breeds (L; n=39)	16	BTrectum=0.796(BTear)+21.07	0.879	0.532
	17	BTrectum=0.592(BTear)+0.193(STrectum)+21.50	0.802	0.621
Giant Breeds (XL; n=3)	-	No variables were entered into the equation		
Coat type (n=380; AKC, 2020)	18	BTrectum=0.293(STrectum)+70.43	1.198	0.339
Short hair (n=204)	19	BTrectum=0.200(STrectum)+0.312 (BTear)+49.01	1.095	0.450
Long hair (n=176)	20	BTrectum=0.560(BTear)+44.70	1.070	0.418
	21	BTrectum=0.372(BTear)+0.189(STrectum)+44.06	0.978	0.516
Five-scale body condition score (n=380) [17]				
1 (n=12)	22	BTrectum=0.097(STpinna)+90.02	1.113	0.366
1.5 (n=2)	-	No variables were entered into the equation.		
2 (n=21)	23	BTrectum=0.291(SThip)+72.09	1.055	0.557
	24	BTrectum=0.303(SThip−0.086(STfootpad)+78.72	0.967	0.647
	25	BTrectum=0.267(SThip)−0.108(STfootpad)+0.117(STear)+72.39	0.796	0.774
2.5 (n=49)	26	BTrectum=0.274(STrectum)+72.41	1.019	0.287
	27	BTrectum=0.271(STrectum)−0.075(SThip)+79.71	0.967	0.372
	28	BTrectum=0.235(STrectum)−0.075(SThip)+0.202(BTear)+63.17	0.930	0.431
3 (n=183)	29	BTrectum=0.322(STrectum)+67.66	1.039	0.430
	30	BTrectum=0.221(STrectum)+0.295(BTear)+48.55	0.958	0.519
	31	BTrectum=0.243(STrectum)+0.311(BTear)−0.035(STfootpad)+47.96	0.942	0.538
3.5 (n=50)	32	BTrectum=0.652(BTear)+35.40	1.233	0.497
	33	BTrectum=0.499(BTear)+0.144(STrectum)+36.03	1.121	0.593
4 (n=42)	34	BTrectum=0.224(STrectum)+77.62	0.970	0.283
	35	BTrectum=0.148(STrectum)+0.241(BTear)+61.42	0.925	0.365
4.5 (n=4)	-	No variables were entered into the equation.		
5 (n=28)	37	BTrectum=0.571(BTear)+43.91	0.953	0.413

SEE=Standard error of estimation, BT=Body temperature and ST=Surface temperature, SE=Standard error

An equation for giant breeds was not generated because of the low sample size. High coefficients of determination were found for small (R^2^=0.582) and large breeds (R^2^=0.532), followed by toy (R^2^=0.391) and medium breeds (R^2^=0.197). For coat type, BTear or STrectum were considered as first predictors for dogs with long or short hair, respectively. The coefficient of determination from the long hair equation (R^2^=0.418) was higher than that from the short hair equation (R^2^=0.339). Regarding the BCS, the equations of BCSs 1.5 and 4.5 were not established because of the low sample size. BCS 2 had the highest coefficient of determination (R^2^=0.557), followed by 3.5 (R^2^=0.497), 3 (R^2^=0.430), 5 (R^2^=0.413), 1 (R^2^=0.366), 2.5 (R^2^=0.287), and 4 (R^2^=0.283). All predictors in the equations were positive to BTrectum, except STfootpad (equation numbers 3, 6, 24, 25, and 31), SThip (equation numbers 27 and 28), and STpinna (equation number 15). The EnvironTemp and humidity were not included in any equation.

**Figure-1:**
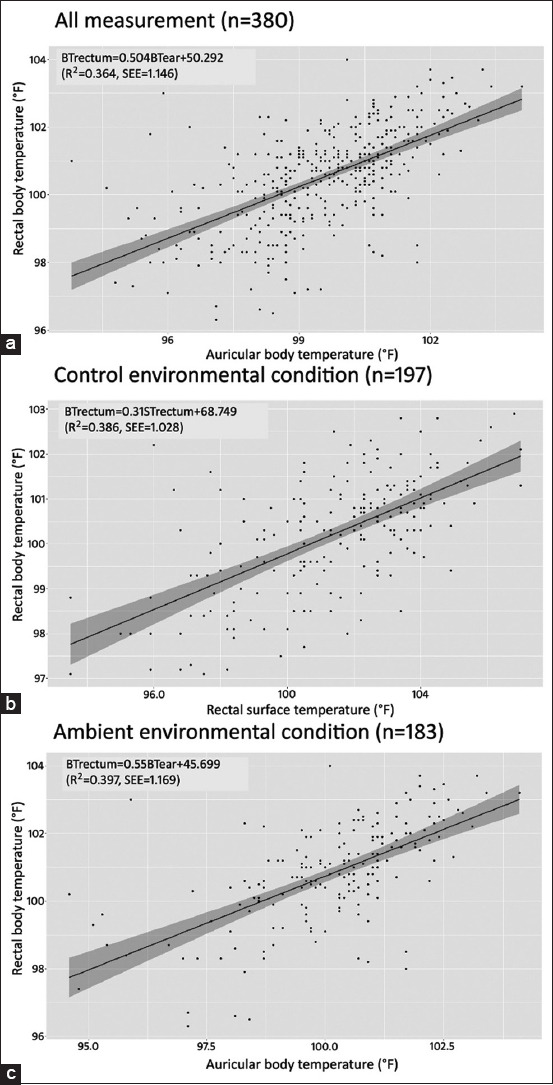
Scatterplots of auricular body, rectal surface or auricular body temperature versus rectal body temperature (°F) from all measurement data (a), control environmental condition group (b) or ambient environmental condition group (c), respectively.

**Figure-2:**
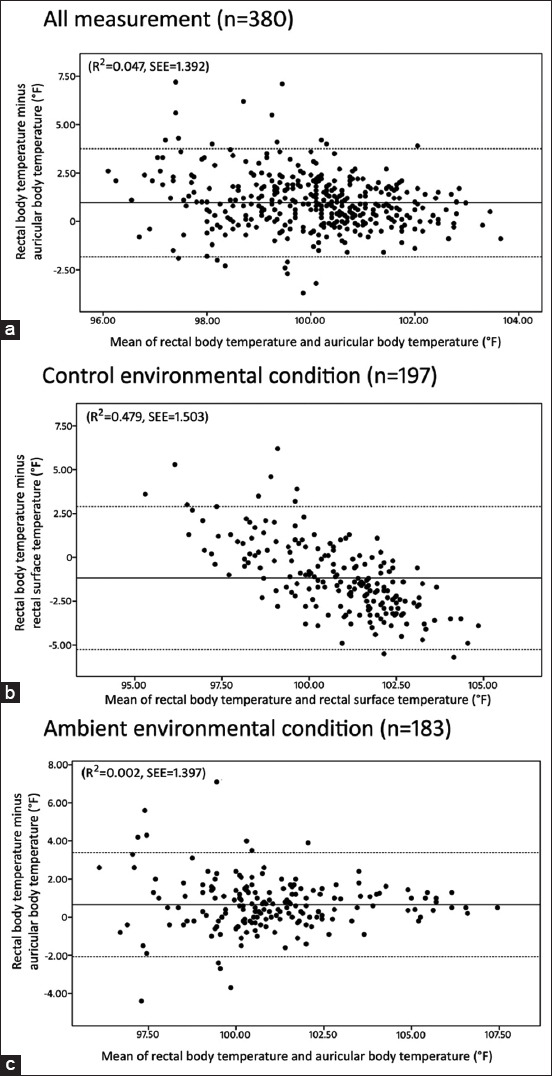
Bland-Altman plot of difference between rectal body temperature and ear body, rectal surface or ear body temperature (°F) versus average of these measures from all measurement data (a), control environmental temperature group (b), and ambient environmental temperature group (c), respectively.

The scatterplots and Bland-Altman plots of BTear or STrectum versus BTrectum from all measurement data and environmental conditions are shown in Figures-1 and 2, respectively. For the Bland-Altman plot, the average and standard deviation values of the differences between BTrectum and BTear, STrectum, or BTear versus the average of these measurements from all measurement data, CT and AT, were 0.97±1.42, −1.18±2.08, and 0.66±1.40, respectively.

## Discussion

### Using auricular or ST as a predictor of rectal temperature

Most ST values were lower than BTrectum, which is in agreement with the previous studies [8]. The emission of infrared radiation from the tympanic membrane receiving heat from the carotid artery, which passes the hypothalamus, was measured as BTear by an auricular thermometer [2,3]. Based on this idea, BTear can be used as an alternative temperature measurement site in humans because the probe of the auricular thermometer can be placed close to the tympanic membrane. However, humans’ ear canal anatomy is different from that of companion animals. In the previous studies, BTear has been investigated as an alternative indicator to estimate rectal temperature in dogs [2-4,6,7] and cats [13] because BTear measurement was easier to take than BTrectum. In dogs and cats, the ear canal has an L shape, making it impossible to place the probe of the auricular thermometer close to the tympanic membrane [3], which resulted in significantly lower BTear compared to BTrectum values, which have also been observed in other studies [3,7]. Moreover, BTrectum was measured inside the rectum, which is better to conserve temperature than the ear canal, which loses temperature by convection. For these reasons, BTear was not identical to BTrectum. However, correlations between BTear and BTrectum have frequently been reported [2-4,6,7]. Moderate correlations have been reported for dogs (r=0.62–0.74, p<0.05; [2]) and cats (r=0.62, p<0.05; [13]); however, some studies have found weak correlations in dogs (r=0.30–0.43 [4] and 0.34–0.37, p<0.05; [6]). Animal signalments, health status, environmental conditions, and study designs were most likely responsible for the differences in the correlation coefficients among the studies. A moderate correlation was found using similar animal signalments (age, breed, body weight, and BCS), health condition, living conditions, and several repeat measurements on the same animals [2,13].

On the other hand, animals in experiments with weak correlations were received by occasional visiting to animal hospital leading to large variation in signalments and health conditions, although animals with otitis were excluded from the studies [4,6]. Interestingly, large variations in BCS (1–5), coat type (long vs. short coat type), breed (28 breeds), size, and environmental condition were observed in this study, with some moderate correlations (r=0.61; p<0.01). This might be a consequence of the high number of animals in the current study (n=380) compared to the studies of Wiedemann *et al*. [6] and Sousa *et al*. [4], with only 53 and 88 dogs, respectively. Therefore, larger datasets can result in higher correlation. However, a decrease in error from the diversity of animal signalments, health status and environmental conditions could be considered. Differences based on the body side of the animal are not reported because, in dogs, there are no anatomical differences between left and right ear, ear canal, and blood supply [7]. However, a slightly higher coefficient of correlation has been found in the left compared to the right ear [6]. Based on the moderate correlation with BTrectum, BTear can be used as an alternative temperature measurement. However, this measurement technique is still labor-intense and induces stress in the animals, and ST might be more suitable.

ST was measured through infrared radiation, and the measurement sites were the major factors influencing prediction accuracy [2,3]. The posterior border of the eyelids and the lacrimal caruncle present large numbers of capillary beds, facilitating the measuring of ST at the eyes to predict BTrectum in humans, cattle, and dogs [2]. However, STeye was poorly correlated to BTrectum (r = 0.38, p<0.001), whereas BTear presented a moderate correlation (r = 0.62, p<0.001; [2]). We assume that this inaccuracy of BTeye is a result of heat loss by convection from the eye surface through wind, whereas BTear and BTrectum, which are taken inside the body, are more constant. However, this hypothesis should be further investigated. In a previous study, a temperature-sensing microchip was placed in the subcutaneous layer at an interscapular, lateral aspect of the shoulder, and sacral region to compare measurements with BTear and BTrectum in dogs [3]. Subcutaneous temperature in all measurement sites was not a good indicator to predict core temperature, most likely because of the variations in subcutaneous fat, environmental conditions, type of hair coat, and BCS [3]. The subcutaneous temperatures at the surface of the body could be considered similar to ST.

On the one hand, a weak correlation of STshoulder (r = 0.14, p<0.01) with SThip (p>0.05), compared to BTrectum, was observed in our study. Similar to STeye, the large temperature loss might have been the cause. In this sense, large outer body surface areas are not suitable sites for temperature measurement in dogs, as dogs also lose heat via the footpads, which are generally in contact with the surface, and STfoodpad was not a good indicator in this study [19]. The hairless area, with a large circulation of peripheral blood flow, has been considered as a measurement site in other animal species [19]. Axillary and inguinal areas were measured in this study as the hairless areas with blood circulation underneath, but a weak correlation with BTrectum was observed. These two areas were not the major sites of heat loss in dogs, making them unsuitable for temperature measurement. As described above, BTear was a good indicator to predict BTrectum, and therefore, STear and STpinna were measured at the ear canal and the pinna. The obtained correlation was weak, most likely because of the long distance between the measurement sites and the tympanic membrane when compared to BTear. In this study, STrectum was measured, which was neglected in most previous studies, and there was a moderate correlation between STrectum and BTrectum (r = 0.61, p<0.01). Although STrectum was not selected as a primary indicator for the equation based on the stepwise technique from all measurement data (Eq. 1), the R^2^ of STrectum (0.386, Eq. 4) was nearly the same as that of BTrectum (R^2^ = 0.364, Eq. 1), most likely because of the hairless area close to the rectum. Moreover, in most dogs, the anal area is covered by the tail, which also prevents heat loss by convection. However, STrectum could be difficult to measure by automatic infrared image analysis as the tail usually covers this area, although it requires less restraint than the measurement of BTear. Therefore, STrectum is a good indicator for the prediction of BT, similar to BTear.

### Factors influencing the prediction equations

Air conditioning reduces the temperature by lowering the humidity; therefore, in our study, the humidity at CT was lower than that at AT. As this study was performed in the summer season in Thailand, the AT group of dogs was subjected to high temperatures. Basal metabolism continuously generates energy and heat in animals, and excess heat needs to leave the body to maintain a normal temperature range [1]. The difference between the ST of animals and the environmental temperature is correlated to heat elimination [19]. Our study observed a lower heat elimination in dogs at AT, resulting in a higher BTrectum and other measurement parameters. Interestingly, Environ Temp and humidity, which represent the environmental conditions, were not part of any prediction equation, which indicates that dogs are not largely influenced by environmental temperatures [1,19].

Based on the study results, STrectum was a good predictor for BTrectum of dogs under CT. We assume that BT is maintained by largely avoiding heat loss by convection because the tail largely covers the anal area at lower temperatures (CT). Therefore, STrectum was measured after lifting the tails to obtain higher accuracy. On the other hand, BTear was more suitable to predict BTrectum of dogs under AT than STrectum. The tails did not cover the anal area at high temperatures, resulting in heat loss, making STrectum an unreliable measurement. However, these hypotheses should be confirmed by further studies.

Small animals contain a larger body surface area-to-body mass ratio, with higher metabolic heat production and faster heat loss from the surface area when compared to large animals [7,19]. For example, Labrador Retrievers (large breed) have a higher BT than Beagles (small breed; [2]), although another study found the opposite pattern [7]. In addition, BTear is generally not influenced by body size [7]. This variation could be due to several factors such as health condition, ambient temperature, and coat type [19]. Moreover, the different characteristics of dog breeds could be another factor influencing these variations. Interestingly, STInguinal, a hairless area with peripheral circulation, was a predictor for medium-size breeds, but a low coefficient of determination was obtained when comparing the values to those of other breed sizes using BTear or STrectum as predictor. Therefore, it cannot be concluded that breed by size influences the accuracy of the prediction equation. Hair, as an insulator, prevents heat loss from animals [19], with long hair providing better protection. In a previous study, the highest ST was observed in the lateral area of short-coat dogs, followed by curly ones and double coat ones [16]. Therefore, the accuracy of the prediction equation based on a long hair coat was higher than that of the equation based on short hair. The subcutaneous fat layer is a temperature insulator and correlated to the BCS [19]. However, the BCS had no clear impacts on the accuracy of the prediction equation, based on the results of this study. Based on our results, there is no universal prediction equation for all breeds, characteristics and conditions of dogs. In other studies, higher coefficients of correlation or prediction accuracies were obtained when using BTear and/or ST to predict BTrectum or BT in livestock animals [5,11,12] compared to the values found in our study and in the previous ones [3,13,14]. For livestock, similarities in breed, characteristics and housing, in contrast to companion animals, might have caused the higher prediction accuracy. In this sense, higher accuracy could be obtained when looking at animals of similar breeds and similar characteristics and conditions. However, this should be further investigated.

## Conclusion

The BTear was a good predictor of BTrectum based on overall data and under ambient temperatures, whereas STrectum was a good predictor under controlled temperatures. Environmental condition, size, coat type, and BCS influenced the accuracy of the prediction equation, and the specific equation must, therefore, be selected based on these factors. In clinical practice, BTear or STrectum can be used to predict BTrectum using the equation from this study. The limitation of this study is the accuracy of the prediction equation, which can be improved by collecting a higher number of data points. To further increase the accuracy of the prediction equation, other influencing factors need to be investigated.

## Authors’ Contributions

CL and AK: Conceptualization. AK: Methodology. SA and AK: Validation. YL, NT, VC, PN, and SA: Investigation. SA and AK: Data curation. SA and AK: Writing - original draft preparation. YL, NT, VC, PN, SA, PC, CL, and AK: Writing-review and editing. AK: Project administration. All authors read and approved the final manuscript.
